# Excitatory and inhibitory STDP jointly tune feedforward neural circuits to selectively propagate correlated spiking activity

**DOI:** 10.3389/fncom.2014.00053

**Published:** 2014-05-07

**Authors:** Florence I. Kleberg, Tomoki Fukai, Matthieu Gilson

**Affiliations:** Lab for Neural Circuit Theory, RIKEN Brain Science InstituteWako, Japan

**Keywords:** STDP, spike-timing, plasticity, inhibition, disynaptic, correlation, excitation–inhibition balance

## Abstract

Spike-timing-dependent plasticity (STDP) has been well established between excitatory neurons and several computational functions have been proposed in various neural systems. Despite some recent efforts, however, there is a significant lack of functional understanding of inhibitory STDP (iSTDP) and its interplay with excitatory STDP (eSTDP). Here, we demonstrate by analytical and numerical methods that iSTDP contributes crucially to the balance of excitatory and inhibitory weights for the selection of a specific signaling pathway among other pathways in a feedforward circuit. This pathway selection is based on the high sensitivity of STDP to correlations in spike times, which complements a recent proposal for the role of iSTDP in firing-rate based selection. Our model predicts that asymmetric anti-Hebbian iSTDP exceeds asymmetric Hebbian iSTDP for supporting pathway-specific balance, which we show is useful for propagating transient neuronal responses. Furthermore, we demonstrate how STDPs at excitatory–excitatory, excitatory–inhibitory, and inhibitory–excitatory synapses cooperate to improve the pathway selection. We propose that iSTDP is crucial for shaping the network structure that achieves efficient processing of synchronous spikes.

## 1. Introduction

Activity-dependent plasticity of synaptic connections between neurons is crucial for cortical circuit development and memory (Böhme et al., 1993; Hensch et al., 1998). Spike-timing-dependent plasticity (STDP) describes the change in synaptic weights where long-term potentiation (LTP) and long-term depression (LTD) depend on the precise timing of presynaptic and postsynaptic action potentials. STDP has been observed for excitatory glutamatergic synapses in a great diversity of brain structures, such as the hippocampus (Magee and Johnston, 1997; Bi and Poo, 1998; Debanne et al., 1998), the cerebellum of the electric fish (Bell et al., 1997), the neocortex (Markram et al., 1997; Sjöström et al., 2001), and the optic nerve in Xenopus (Zhang et al., 1998). An extensive body of theoretical work has uncovered many interesting properties of excitatory STDP (eSTDP): it can select input pathways based on their spike-time correlation (Kempter et al., 1999; Song et al., 2000; Gjorgjieva et al., 2011), it can generate a stable distribution of weights (van Rossum et al., 2000; Gütig et al., 2003; Gilson and Fukai, 2011), it can perform selection of phase-locking in population firing (Gerstner et al., 1996; Senn and Buchs, 2003), it favors the emergence of functional neuronal assemblies (Izhikevich et al., 2004; Clopath et al., 2010), it stabilizes slow oscillations in recurrent networks (Kang et al., 2008) and it allows for rewiring of connections in the developing visual cortex (Song and Abbott, 2001; Senn and Buchs, 2003; Young et al., 2007).

There is also evidence for STDP at inhibitory GABAergic synapses, or iSTDP, (Woodin et al., 2003; Haas et al., 2006; Kodangattil et al., 2013). However, our understanding of the mechanistic implications of iSTDP remains limited, in spite of the key role of inhibition in signal processing in the cortex (van Vreeswijk and Sompolinsky, 1996; Anderson et al., 2000; Wehr and Zador, 2003; Haider et al., 2006, 2013; Maffei et al., 2006; Rudolph et al., 2007). Considering the abundance of inhibitory interneurons, e.g., in the cortex (Markram et al., 2005), remarkably few types have been tested for the plastic properties of their synapses. Theoretical knowledge of the dynamics and functional implications of iSTDP is also rudimentary, although interest in this direction has increased recently. Extending a simple homeostatic control of firing rate, iSTDP can generate a balance between excitatory and inhibitory inputs onto a neuron (Vogels et al., 2011). In addition, unspecific, but sufficiently strong inhibition developed by iSTDP can enhance competition between excitatory synapses subject to eSTDP (Luz and Shamir, 2012). Interestingly, neither experimental nor theoretical approaches provide a consensus for the shape of the iSTDP learning window, in contrast to eSTDP for which the temporally Hebbian nature (LTP for pre-post pairing, LTD for post-pre pairing) is observed and addressed in the vast majority of cases.

The present paper aims to compare the effect of distinct iSTDP window shapes on the structure of synaptic weights, and endeavors to clarify the role of iSTDP in tuning neuronal responses. Previous studies have shown the precise timing of spikes to convey an important part of information about stimuli in sensory pathways (Riehle et al., 1997; Jackson et al., 2003; Maldonado et al., 2008; Kilavik et al., 2009; Putrino et al., 2010). Moreover, neurons are sensitive to precise timings of spikes (Rossant et al., 2011). In this context of neural temporal coding, we examined the transmission of temporally correlated spikes in a feedforward circuit equipped with eSTDP and iSTDP. Such neural architectures with joint feedforward excitation and inhibition have been found in various brain structures (Buzsaki, 1984; Davis et al., 1996). We incorporated in our model an important property of the afferent inputs observed experimentally in many feedforward neural architectures: inhibition is delayed compared to excitation with a short time lag (Pouille and Scanziani, 2001; Wilent and Contreras, 2004; Gabernet et al., 2005; Silberberg and Markram, 2007; Tan et al., 2008; Stokes and Isaacson, 2010), which allows for precise temporal gating (Kremkow et al., 2010). We found that iSTDP with specifically anti-Hebbian properties enforces a balanced structure in the synaptic weights, which supports efficient processing of near-coincident spikes.

## 2. Results

We examined the joint development of excitatory and inhibitory synapses subject to STDP in a feedforward circuit. We consider two circuit architectures. First, for a single neuron with direct excitatory and inhibitory inputs, we examine how the shape of the iSTDP window affects the evolution of synaptic weights. Since there is no current consensus about a single type of iSTDP (Woodin et al., 2003; Haas et al., 2006; Kodangattil et al., 2013), this comparison allows us to link the shape of iSTDP learning windows to their functional implications. Second, we examine the recruitment of interneurons in a more realistic circuit with monosynaptic excitation and disynaptic inhibition. In both cases, we focus on how the emerging weight structure tunes the propagation of spike volleys in the circuit.

### 2.1. Theoretical prediction of weight specialization depending on iSTDP window

**Figure 1:**
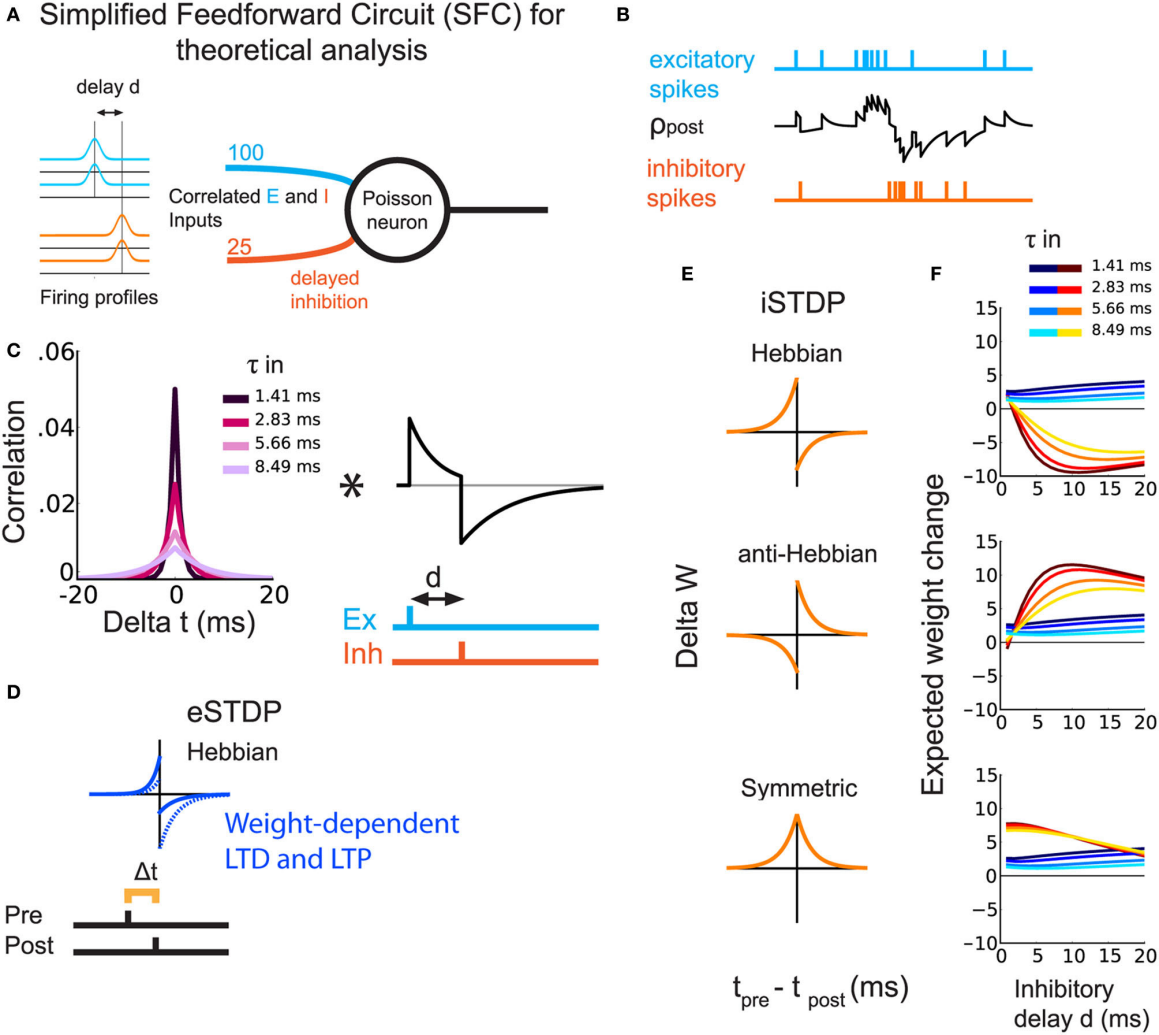
**Theoretical prediction of weight update for different types of iSTDP windows**. **(A)** Schematic representation of the SFC. The Poisson neuron receives excitatory (light blue) and inhibitory (orange) inputs. All inputs are correlated with equal strength and a delay *d* for inhibition. **(B)** Example of the postsynaptic firing rate (black trace) in response to excitatory (light blue) and inhibitory (orange) spikes. **(C)** Left: Temporal cross-correlogram for a pair of similar input spike trains, excitatory or inhibitory. Lighter colors correspond to larger correlation spread τ_in_. Right, EPSP-IPSP shape including *d*. The asterisk indicates the convolution. **(D)** eSTDP window. The left (right) part in the window indicates the presynaptic spike occurring before (after) the postsynaptic spike. **(E)** The three iSTDP learning windows: Hebbian, anti-Hebbian, symmetric. The expected weight change is the double convolution between the left and right figures in **(C)** and the window functions in **(E)**. **(F)** Expected mean weight changes by analysis of a Poisson rate model for excitatory and inhibitory weights with simultaneous eSTDP and iSTDP. The plots correspond to the three iSTDP windows in **(E)**. Warm colors represent inhibitory weights and cold colors excitatory weights. Lighter colors correspond to lighter colors in **(C)**, namely larger τ_in_.

In order to study the weight dynamics for different iSTDP learning windows, we consider a simplified feedforward circuit (SFC) that consists of a single postsynaptic Poisson neuron excited by excitatory and inhibitory spike trains (Figure 1A). Following experimental observations, excitatory and inhibitory inputs have correlated spiking activity (Okun and Lampl, 2008). In addition, inhibition arrives with a delay *d* (Okun and Lampl, 2008; Atallah and Scanziani, 2009). The inhibitory delay mimics a disynaptic pathway, as compared to monosynaptic excitation (Figure 1B). The temporal correlations between spikes trains in Figure 1B are governed by the time constant τ_in_ (Figure 1C). All Synapses are plastic.

Excitatory weights are modified by a temporally Hebbian eSTDP rule (Gilson and Fukai, 2011), corresponding to the blue learning window in Figure 1D: a presynaptic spike preceding a postsynaptic spike leads to potentiation. The eSTDP update includes log-STDP weight dependence, which produces a long-tailed distribution of weights (Gilson and Fukai, 2011). For every pair of a pre- and a postsynaptic spikes, the weight *w*_*e*_ is modified by a quantity that depends on the current value of *w*_*e*_ and the spike-time difference Δ *t* = *t*_pre_ − *t*_post_:

(1) $$\Delta w_{\text{e}}=W_{\text{e}}(\Delta t,w_{\text{e}})=\{\begin{array}{l} \eta_{\text{e}}\exp\left(\frac{\Delta t}{\tau_{\text{LTP}}^{\text{e}}}\right)a_{\text{LTP}}\exp\text{​}\left(-\frac{C_{\text{LTP}}w_{\text{e}}}{w_{\text{0}}}\right)\text{ for }\Delta t<0,\\ \eta_{\text{e}}\exp\text{​}\left(-\frac{\Delta t}{\tau_{\text{LTD}}^{\text{e}}}\right)a_{\text{LTD}}\exp\text{​}\left(\frac{\log(1+w_{\text{e}}/w_{0}C_{\text{LTD}})}{\log(1+C_{\text{LTD}})}\right)\text{for}\Delta t>0. \end{array}$$

The time constants τ^e^_LTP_ = 17 ms τ^e^_LTD_ = 34 ms and coefficients *a*_LTP_ = 1 and *a*_LTD_ = −0.5 determine the shape of the eSTDP window. η_*e*_ is the learning rate. The log-style weight dependence scales the LTD curve and ensures a stable fixed point at *w*_0_ = 0.065 for uncorrelated inputs; *C*_LTD_ = 5 enforces sufficiently strong competition between the incoming weights onto a given neuron. An exhaustive eSTDP parameter list is given in Table 1.

**Table 1:** **General STDP parameters**.

**STDP parameters: SFC**	**Description**	**Value**
W_*e*_, *W*_*i*_	eSTDP or iSTDP window	
τ^e^_pre_,τ^e^_post_	eSTDP window time constants	34 ms, 17 ms
τ^i^_pre_, τ^i^_post_	iSTDP window time constants	30 ms
–	Excitatory start-up weights	random U [0, *w*_0_ × 3]
–	Inhibitory start-up weights	0
*w*_0_	Equilibrium weight for eSTDP	0.065
η_e_	Excitatory learning rate	*w*_0_ × 0.39
η_i_	Inhibitory learning rate	0.075
α	inhibitory presynaptic single-spike contribution	0.2
C_*LTD*_	LTD scaling for eSTDP	5
C_*LTP*_	LTP scaling for eSTDP	50
**STDP PARAMETERS: FFC**		
*w*_0_	Equilibrium weight for eSTDP onto output neuron	0.08
η_e_	Excitatory learning rate	*w*_0_ × 0.78
η_i_	Inhibitory learning rate	0.02
–	Excitatory start-up weights	random U [0, 1]
–	Excitatory-to-Inhibitory start-up weights	random U [0, 1]
–	Inhibitory start-up weights	1
τ^i^_pre_, τ^i^_post_	iSTDP window time constants	20 ms
*w*^i^_0_	Equilibrium weight for eSTDP onto interneurons	*w*_0_ × 2
–	Excitatory start-up weights onto interneurons	random U [0, 1]

Table listing all eSTDP and iSTDP parameters, with the exception of the iSTDP window parameters (Table 2). The notation “random U [a, b]” denotes a random distribution between a and b.

For inhibitory synapses, we test three types of additive iSTDP windows, shown in orange in Figure 1E:

- Hebbian (Haas et al., 2006; Luz and Shamir, 2012; Kodangattil et al., 2013), with pre-post LTP;
- Anti-Hebbian, with post-pre LTP;
- Symmetric (Vogels et al., 2011), with which LTP occurs for pre-post and post-pre spike pairings.

For every spike pair the inhibitory weight is updated with

(2) $$\Delta w_{\text{ i}}=W_{\text{ i}}(\Delta t)=\{\begin{array}{l} \eta_{\text{i}}p\exp\text{​}(-\frac{\Delta t}{\tau_{\text{post}}^{\text{i}}})\text{for}\Delta t>0,\\ \eta_{\text{i}}q\exp\text{​}(\frac{\Delta t}{\tau_{\text{pre}}^{\text{i}}})\text{for}\Delta t<0. \end{array}$$

The right and left sides of the iSTDP window can be either LTP or LTD depending on the sign of *p* and *q*, respectively. Table 2 lists the values of *p* and *q* for the three windows employed in the theoretical model and in the simulations. For all iSTDP window types, total LTP exceeds total LTD; for anti-Hebbian, Hebbian, and symmetric (corrected), the difference LTP—LTD is set equal. Additionally, τ^*i*^_pre_ = τ^*i*^_post_ for all iSTDP.

**Table 2:** **iSTDP window parameters**.

**iSTDP type**	**Hebbian**	**anti-Hebbian**	**Symmetric**	**Symmetric (equal LTP/LTD)**
*p*	−1	1.5	1.5	0.25
*q*	1.5	−1	1.5	0.25

Table listing the different iSTDP windows. p and q indicate the amplitude of the right and left side of the iSTDP window, respectively.

To stabilize iSTDP, each inhibitory weight is decreased by a small amount α for every presynaptic spike (Vogels et al., 2011), independently of the iSTDP contribution.

(3) $$W_{i}\to W_{i}-\eta_{i}\alpha$$

Our aim is to show the emergence of balance between excitation and inhibition through eSTDP and iSTDP. By balance we mean the simultaneous increase of excitatory and inhibitory weights, or weight balance, as opposed to increase of excitatory weights without increase of inhibitory weights. Balance known as the cancelation of currents onto a neuron (e.g., Vogels et al., 2011) can but need not follow from weight balance. Unless otherwise stated, balance in this study means weight balance. Using our analysis based on the Poisson neuron model (Materials and Methods), we evaluate the *expected change* in mean synaptic strengths for both sets of weights. The weight update is determined by the interplay of the iSTDP window, the input spike-time cross-correlograms and the postsynaptic response (EPSPs+IPSPs; Figure 1C). All expected weight changes in Figure 1F (and in subsequent sections, weights themselves) are shown after division by the excitatory equilibrium weight *w*_0_, given in Table 1. The influence of the inhibitory delay *d* on the expected change in excitatory weights is weak—slight increase when *d* becomes larger—and does not depend much on the iSTDP learning window (Figure 1F, curves in cold colors). However, *d* affects the evolution of inhibitory weights, as shown by the curves in hot colors in Figure 1F. For Hebbian iSTDP, inhibitory weights decrease with a stronger effect for larger delays (≥5 ms). Conversely, anti-Hebbian iSTDP causes weights to increase. Symmetric iSTDP leads to a potentiation that weakens for large delays.

In all cases, larger values for the input correlation width τ_in_ decrease the effect of both eSTDP and iSTDP (curves in lighter colors in Figure 1F). In fact, Hebbian and anti-Hebbian iSTDP curves exhibit a delay for which the weight change is maximal. That “best” delay increases when τ_in_ is large. Symmetric iSTDP is less affected by τ_in_.

In summary, the simultaneous strengthening of correlated excitatory and inhibitory inputs (i.e., the emergence of balance) should occur when iSTDP has an anti-Hebbian LTP component in this simple circuit (anti-Hebbian and symmetric iSTDP), and when inhibitory input spikes arrive after postsynaptic spikes with a sufficiently large *d* (axonal delay in a feedforward inhibitory circuit).

### 2.2. Emergence of a detailed balance between excitatory and inhibitory weights

**Figure 2:**
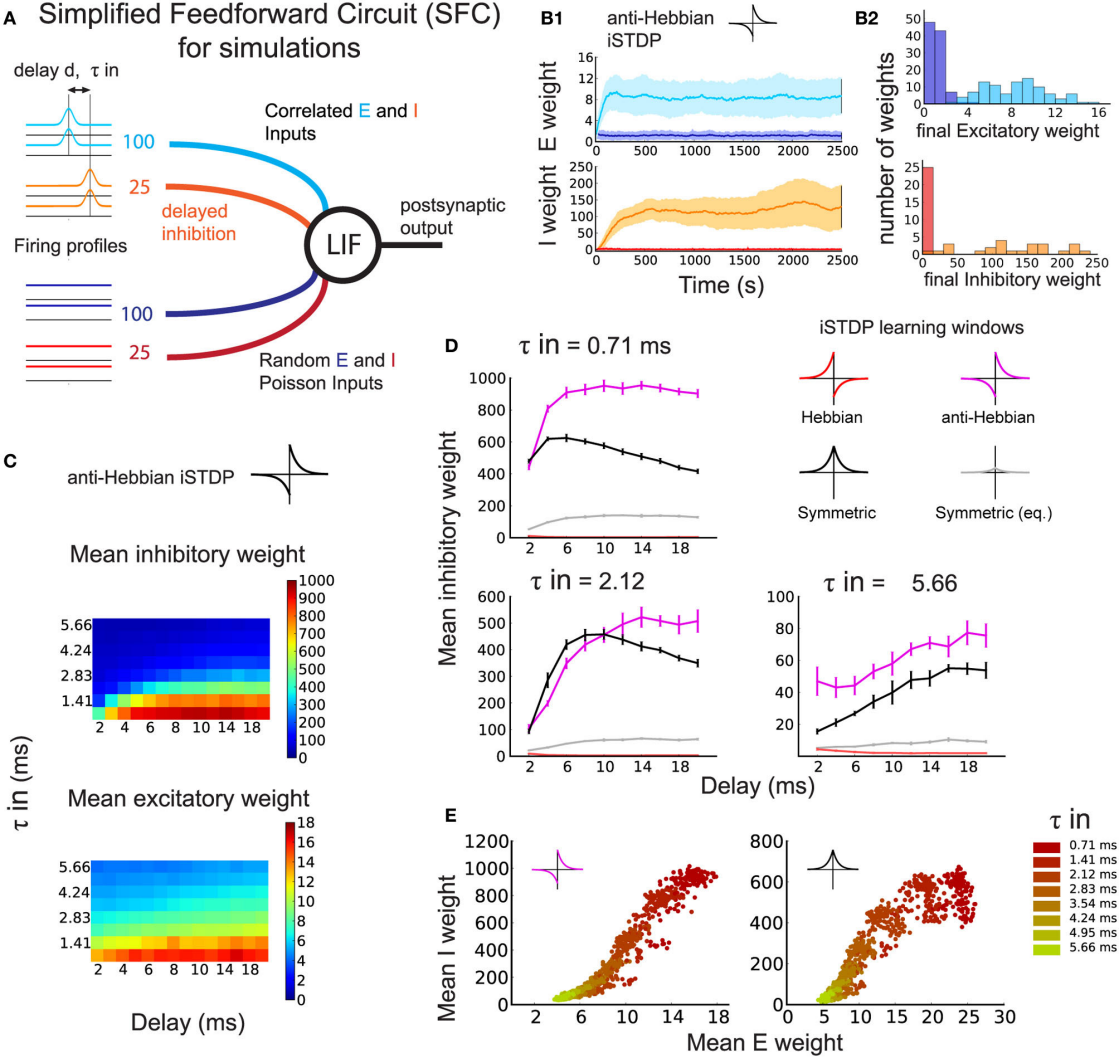
**Development of the synaptic structure induced by simultaneous eSTDP and iSTDP (numerical simulation)**. **(A)** Schematic representation of the Simplified Feedforward Circuit Model (SFC). One leaky integrate-and-fire (LIF) neuron receives four different input groups, of which half have temporal correlations with time constant τ_in_ (light blue and orange) and half are random spike trains (dark blue and red). Spike-time correlations arise from common variation of the firing rate. Inhibition arrives after excitation with a delay *d*. **(B1)** Example of weight evolution over time of a simulation using eSTDP and anti-Hebbian iSTDP. Excitatory weights (top), inhibitory weights (bottom). The delay is *d* = 3 ms, and input temporal precision τ_in_ = 2.12 ms. **(B2)** Histograms of the distribution of all weights at the end of the anti-Hebbian iSTDP simulation shown in **(B1)**. **(C)** Mean inhibitory (top) and excitatory (bottom) weights from the correlated input group after learning. Each pixel corresponds to the average of 10 simulations with inhibitory delay *d* (*x*-axis) and input temporal resolution τ_in_ (*y*-axis). **(D)** Mean inhibitory weight of the correlated inputs after learning with different iSTDP windows: Anti-Hebbian (magenta), Hebbian (red), symmetric (black, gray) for three values of τ_in_. Each plot corresponds to a line in **(C)**. **(E)** Final excitatory and inhibitory weights of the correlated pathway for anti-Hebbian (left) and symmetric (right) iSTDP, shown for all τ_in_ and *d* = 6 ms. One dot represents the mean excitatory and mean inhibitory weight at the end of one trial. Delays are pooled within the same color.

Next, we verify our theoretical predictions for the SFC with simulations using a LIF neuron (Materials and Methods: details of the simulated SFC, Equations 8, 9). In contrast to Figure 1, the SFC in Figure 2A includes a distractor pathway with random, uncorrelated inputs (Figure 2A, dark blue and red lines) besides the correlated inputs (light blue and orange lines).

A typical example of synaptic weight evolution with anti-Hebbian iSTDP is shown in Figure 2B1. The weights from random inputs remain weak (dark blue and red traces), whereas the weights from the correlated excitatory inputs (light blue traces) and inhibitory inputs (orange traces) are strengthened, indicating the development of within-pathway balance, or *detailed balance* (Vogels and Abbott, 2009). In detailed balance, the excitatory and inhibitory inputs to strong weights on a postsynaptic neuron have correlated spike times (or spike rates, Vogels and Abbott, 2009). In contrast, when excitation is balanced with inhibition from a different signal pathway, excitation and inhibition are not necessarily correlated, which we may call *global balance*. Both types of balance will be evaluated in the sections below. The histograms of the final weight distributions of this example show the development of weight structure for excitation and inhibition. Excitatory weights exhibit a long-tail distribution that follows from the log-type weight dependence used for eSTDP (Gilson and Fukai, 2011) (Figure 2B2: top). The distribution of inhibitory weights has a long tail as well (Figure 2B2: bottom), but looks more bimodal for smaller τ_in_ due to increased competition between the weights (not shown).

As in our analytical approach, we compared the effect of different iSTDP windows on the inhibitory weights of an inhibitory–excitatory pathway with correlated spike-times. The comparison of the Hebbian, anti-Hebbian, and symmetric iSTDP windows agrees with the theoretical predictions of expected drift in weights in Figure 1F. Correlated inhibitory weights increase with both symmetric (Figure 2D, black curves) and anti-Hebbian iSTDP (magenta curves). Their final equilibrium value depends on *d* (see also Figure 2C): short delays are preferred only by symmetric iSTDP (Figure 2D, black curves). Anti-Hebbian iSTDP leads to a larger increase in inhibitory weights than symmetric iSTDP for larger delays *d*, and small τ_in_. We also test an additional version of symmetric iSTDP: apart from the window with the same maximal amplitude as Hebbian and anti-Hebbian iSTDP (Figure 1E, bottom; Figure 2D, black curves), we apply a symmetric rule with the same amount of LTP-LTD area (equalized symmetric iSTDP; gray curves). Since this equalized window only leads to very small changes, we conclude that the amplitude of LTP is the crucial factor, not the overall LTP/LTD ratio. Lastly, inhibitory weights vanish to zero with Hebbian iSTDP (red curves). These findings confirm that the neuron first becomes driven by the correlated excitatory inputs through eSTDP; then, when excitatory inputs dictate postsynaptic firing times, correlated inhibitory inputs follow up through iSTDP. The increase in inhibitory weight is determined by *d*, the timing of inhibitory spikes to the excitatory spikes (and therefore to the output spikes), together with the shape of the iSTDP window. Note that we set α such that weights from background inputs remain weak.

The potentiation of excitatory and inhibitory weights with both anti-Hebbian and symmetric iSTDP exhibits a balance between correlated excitation and inhibition, as illustrated in Figure 2E. Stronger excitatory weights induced by eSTDP are counterbalanced by stronger inhibition due to iSTDP. This phenomenon depends on the input correlation precision τ_in_ (smaller values in darker color), but not significantly on the delay *d*. The matching is not linear and depends on the learning window.

In summary, we find that simulations with the LIF neuron confirm the theoretical results with the Poisson neuron. Detailed balance in the weights from the correlated pathway can arise if the iSTDP window is anti-Hebbian or symmetric, but not if it is Hebbian.

### 2.3. Sharpening the neuronal response in the SFC

**Figure 3:**
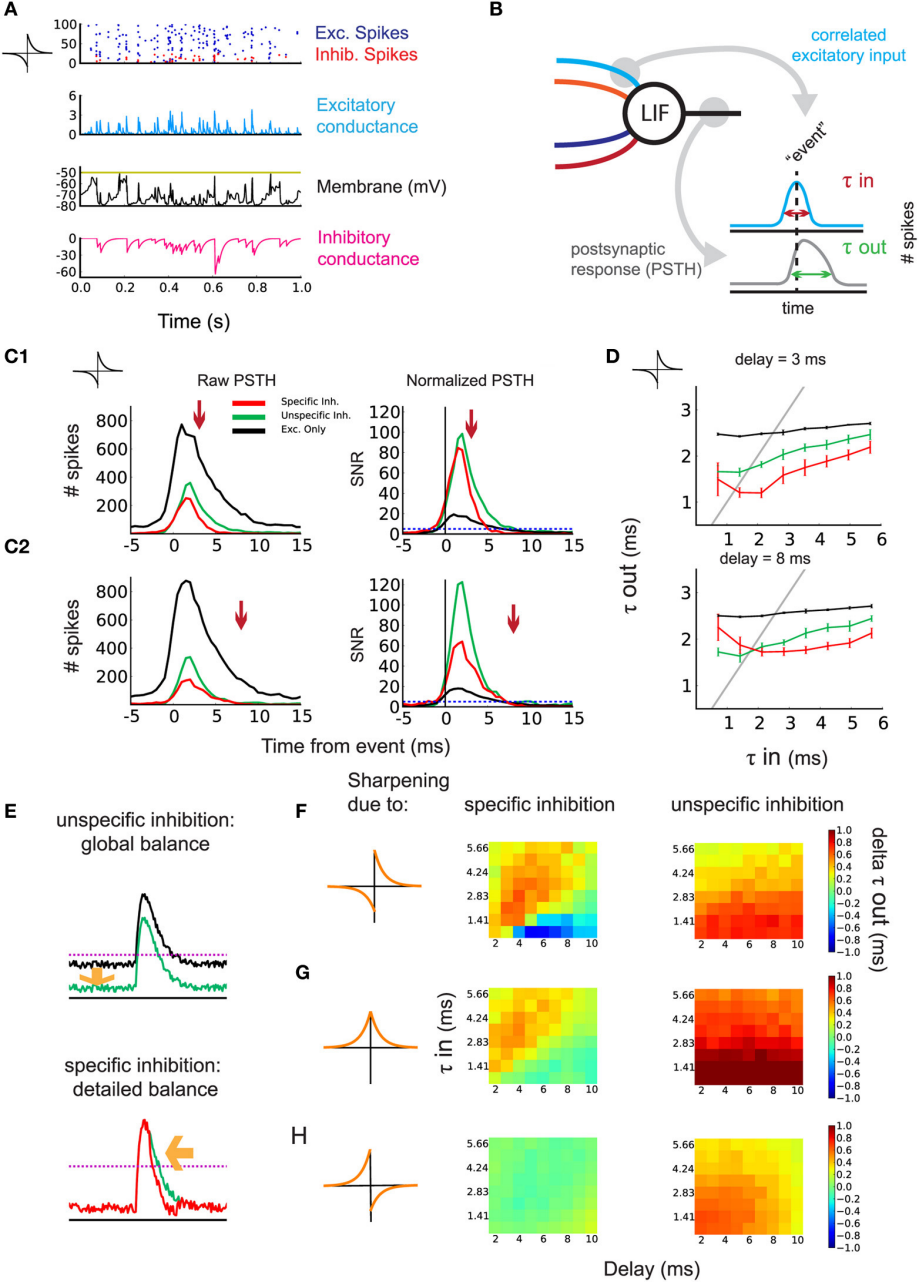
**Sharpening of the postsynaptic response by timed inhibition**. **(A)** Neuronal state after learning. One second activity after training for the simulation in Figure 2B: raster plot of the input excitatory/inhibitory spikes (blue/red), excitatory/inhibitory conductance (blue/magenta), and voltage (black). **(B)** Schematic indicating the construction of the PSTH. Events are detected using the correlated excitatory inputs (blue). Then, postsynaptic spikes that occur in a given window around the event are counted (gray). Response efficiency is evaluated by the temporal width of the PSTH τ_out_. **(C1)** Effect of inhibition on the response of the postsynaptic neuron to correlated events for τ_in_ = 2.12 ms and *d* = 3 ms. Left: example of raw PSTH for postsynaptic spike count. Comparison of detailed balance (red) with the control of global balance (green) and no inhibition (black). The arrow indicates incoming specific inhibition. Right: signal/noise ratio (SNR) obtained by normalizing the PSTHs. **(C2)** Same as in **(C1)** but with *d* = 8 ms. **(D)** Response sharpening for different values of τ_in_ and τ_out_. The gray unit line represents instances where the output width and the input width are equal. Top: *d* = 3 ms. Bottom: *d* = 8 ms. Legend corresponds to **(C)**. **(E)** schematic indicating the effect of detailed balance and global balance on the response shape. **(F)** anti-Hebbian iSTDP learning window and the contribution of detailed and global balance to the sharpening of the response. The difference in τ_out_ is shown for varying *d* (*x*-axis) and the input τ_in_ (*y*-axis). Left: difference in τ_out_ between detailed balance and global balance. Warm colors indicate the response is sharper through detailed balance compared to global balance. Right: difference in τ_out_ between global balance and no inhibition. **(G)** Same as in **(D)** but for symmetric iSTDP. **(H)** Same as in **(D)** but for Hebbian iSTDP, where no detailed balance emerged.

While detailed balance between excitatory and inhibitory weights can arise through anti-Hebbian or symmetric iSTDP (Figure 2), anti-Hebbian iSTDP may increase inhibition to the point where it will dominate over excitation for the postsynaptic neuron, as shown in Figure 3A. This follows partly because we use additive iSTDP, which strongly potentiates inhibitory weights (with our choice of parameters). Whether inhibition dominates (Rudolph et al., 2007) or not, the detailed balance weight specialization underlies the tuning of the SFC function in propagating spike volleys.

As τ_in_ governs the temporal width of input spike volleys, we evaluate τ_out_ for the postsynaptic response (Figure 3B). To do so, we detect volleys whose coincident spikes exceed a threshold as “events.” We then build a peristimulus time histogram (PSTH) of the postsynaptic spikes with respect to the input events (Figure 3B: right). An example for the simulation in Figure 3B with anti-Hebbian iSTDP is shown in Figures 3C1,C2 for two values of *d*.

The PSTH obtained from the simulations with specific inhibition (leading to *detailed balance*; red curve) is compared to two control conditions:

- the inhibitory weights are swapped to obtain unspecific inhibition (leading to *global balance*; green curve)
- the inhibitory inputs are omitted (black curve).

The three conditions are characterized by different mean firing rates. For τ_in_ = 2.12 in the SFC with anti-Hebbian iSTDP, *r*_out_ = 2.21 sp/s for specific inhibition, 2.23 sp/s for unspecific inhibition, and 38.94 sp/s for excitation only.

The difference in response width τ_out_ between specific inhibition and the unspecific inhibition control represents the particular contribution of detailed balance on output response sharpening. Likewise, removing the inhibitory inputs from the circuit and taking the difference with the unspecific inhibition control should reveal the response sharpening due to the general presence of inhibition (global balance). To evaluate the relative change of spiking probability induced by the input stimuli, we normalize the PSTH with respect to the mean postsynaptic firing rate (see Materials and Methods for details). This gives a signal/noise ratio (SNR) for the output spikes following an event in this detection task. As can be seen in Figure 3C (right), both the specific inhibition (red) and unspecific inhibition (green) enhance the SNR. The postsynaptic response is even sharper with the specific inhibition circuit for small delays (Figure 3C1, detailed balance, red curve). This occurs when inhibition is timed with excitation (arrow). For larger *d* as in Figure 3C2, 8 ms, this sharpening vanishes, as inhibition cannot arrive sufficiently early right after excitation. In that case, the performance is closer to that with unspecific inhibition (global balance, green curves). This sharpening is efficient for all τ_in_ = 0.71–5.66 ms, in the range of the delay *d*, as illustrated in Figure 3D. Note that very small delays *d* prevent a proper weight structure from developing with anti-Hebbian iSTDP, thus the sharpening of the response fails (Figure 3D: top: red curve). The principle can be explained by the presence of inhibition lowering the output firing rate, which increases the SNR of the neuron's response (Figure 3E: top). Additionally, precisely timed inhibition coming right after excitation further sharpens the response and improves the SNR (Figure 3E: bottom). Figure 3F summarizes the performance of the sharpening by the emerged detailed weight balance, as compared to the global balance with unstructured inhibition or in the absence of inhibition.

In our model, symmetric iSTDP performed similarly to anti-Hebbian iSTDP (Figure 3G). A small difference is that global inhibition contributed slightly more to the sharpening of the response than the inhibition in detailed balance. The performance is a bit better for anti-Hebbian iSTDP because the weights grow stronger. Lastly, because the weight structure does not develop with Hebbian iSTDP, no significant difference in τ_out_ is observed (Figure 3H: left). Actually, the weights from the random input group are not weakened to zero in the Hebbian case (Supplementary Figure 1), so the unspecific inhibition control condition, in which weights between the two pathways are swapped, leads to a slightly better performance (Figure 3H: right).

We conclude that detailed balance, as achieved by anti-Hebbian and symmetric iSTDP, in combination with Hebbian eSTDP, can lead to the temporal restriction of a postsynaptic response to correlated input spikes. Brief delays in inhibition prove most beneficial for this sharpening, though the exact optimal delay is dependent on the input correlation precision τ_in_.

### 2.4. Recruitment of disynaptic inhibitory pathway with delay selection

**Figure 4:**
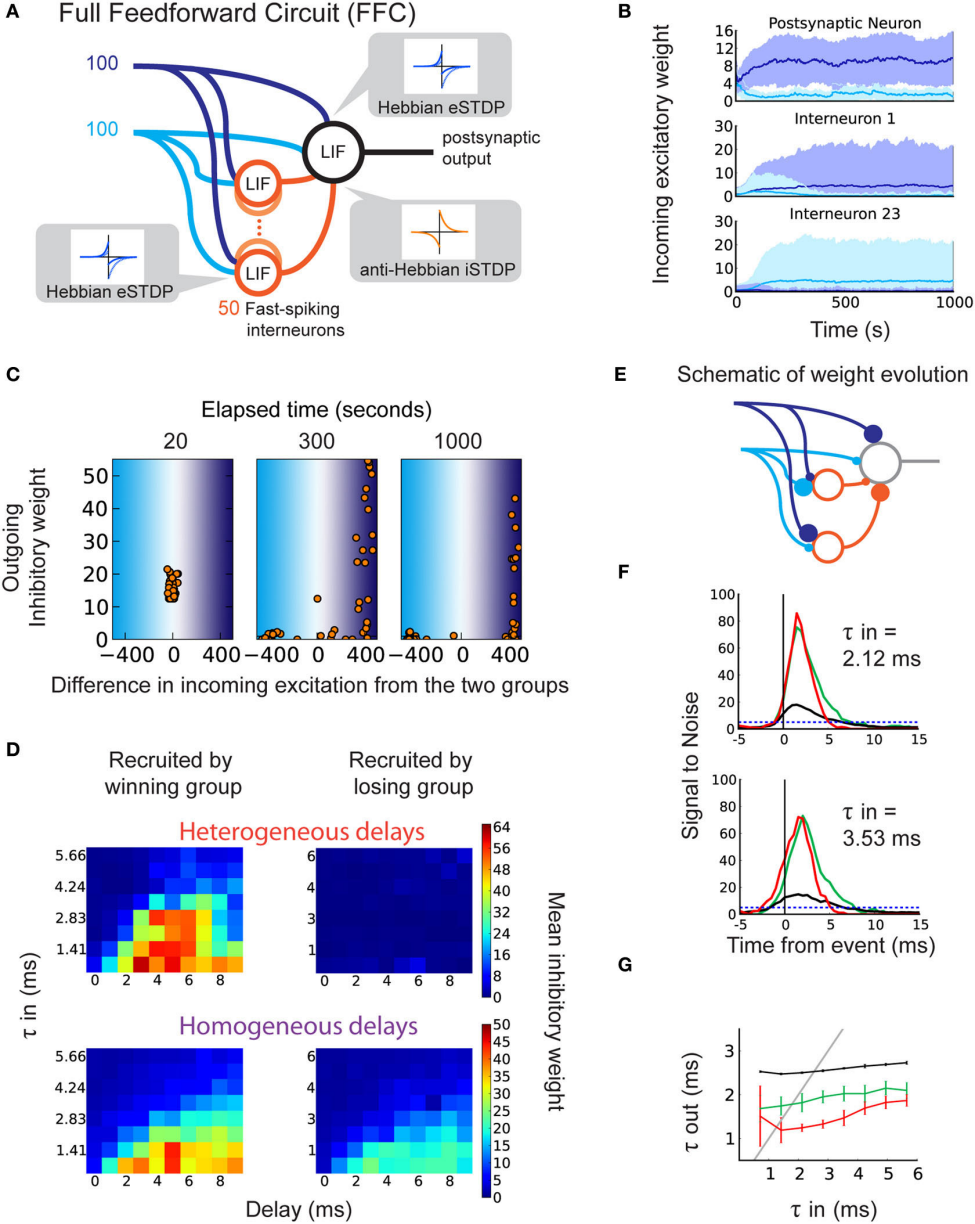
**Selection of delays in a disynaptic pathway by iSTDP**. **(A)** Schematic representation of the full feedforward circuit model (FFC). The postsynaptic neuron receives excitatory input from two correlated groups that compete. Inhibition onto this neuron is provided via 50 fast-spiking interneurons (orange circles). Each interneuron receives inputs from both excitatory groups. The interneurons have axonal delays between 0 and 9 ms. All synapses are plastic, with learning windows shown by the insets. **(B)** The evolution of excitatory weights from the two input groups onto the output neuron (top) and onto two of the 50 interneurons (middle, bottom) in one example trial. Weights from group 1 (dark blue inputs) increase beyond those from group 2 (light blue inputs). Here, group 1 is the “winning group.” Interneuron 1 (23) receives more input from the dark (light) blue group. **(C)** Example of weight evolution onto interneurons and the subsequent change in inhibitory synaptic weights during the simulation (after 20, 300, 1000 s). Each dot represents one of the 50 interneurons. The *x*-axis indicates the difference in total weights between the two input groups onto the interneuron. The right (left) part corresponds to interneurons specializing to the dark (light) blue input group. The *y*-axis indicates the weight of the inhibitory synapse onto the postsynaptic neuron. **(D)** Inhibitory weights after learning depend on the axonal delays of interneurons (*x*-axis) and specialization of their input weights, in the FFC with heterogeneous delays (top; each horizontal line represents an average over 10 simulations), and in the FFC with homogeneous delays (bottom; each square represents 10 simulations). **(E)** Schematic of the recruitment of interneurons and the consequence on the inhibitory weights, leading to detailed balance. **(F)** SNR of the response to correlated events in the FFC with heterogeneous delays. Top: for τ_in_ = 2.12 ms. Bottom: for τ_in_ = 3.54 ms. **(G)** Relationship between τ_in_ and τ_out_ for the FFC model: specific inhibition (red), unspecific inhibition (green), and without inhibitory interneurons (black).

Finally, we explicitly model inhibitory interneurons in our circuit in order to examine how they are recruited in a more realistic architecture. In our FFC model in Figure 4A, two correlated input pathways (dark and light blue) compete against each other. The inhibitory inputs contain heterogeneous axonal delays. Here we focus on anti-Hebbian iSTDP, which proved efficient in developing feedforward inhibition in the previous sections. All excitatory synapses are subject to eSTDP as in Figure 2. In the example simulation in Figure 4B, the excitatory weights onto the output neuron (top) specialize to the dark blue group (“winning group”) at the expense of the light blue group (“losing group”). Note that in general, each group has 50% chance of winning because we use sufficiently competitive eSTDP (Gilson and Fukai, 2011). The inputs onto the interneurons specialize in a similar fashion, as shown for two different examples in Figure 4B (middle and bottom).

After the specialization of excitatory synapses, inhibitory synapses start to become potentiated. We find that the structure in the inhibitory synapses develops only for interneurons that specialize to the same group as the output neuron (Figure 4C: right part of last panel; Figure 4D, top). This is a consequence of the correlation between the spike trains fired by the interneurons that specialize to the winning group, and the output neuron spike train. Conversely, interneurons that specialize to the losing group do not match their spike times to postsynaptic spikes, and their weights remain weak (Figure 4C: left part of last panel). Inhibitory and excitatory inputs onto the output neuron become correlated, making detailed balance possible (Figure 4C, last panel; Figures 4D,E). The use of homogeneous delays in the FFC still achieves detailed balance (Figure 4D, bottom) though the difference in inhibitory weight is smaller. This is because there is no competition between winner-recruited neurons of different delays (Figure 4D bottom, left), and loser-recruited neurons can more easily adjust their firing times to postsynaptic firing if they receive a small amount of input from the winning group (Figure 4D bottom, right).

Importantly, LTP in the inhibitory weights depends on the axonal delay of their interneurons, in a similar manner as the SFC (Figure 4D: left; Figure 2C: top). For broader input spike volleys with larger τ_in_, short delays are *not* selected by anti-Hebbian iSTDP. This ensures that inhibition will not cut off the output response before sufficiently many inputs are integrated. Similarly, late-arriving inhibition does not affect the sharpening of the response, therefore there is no need for its weight to be increased for this function. The adequately timed inhibition that follows excitation results in a sharper response to correlated events in the FFC (Figures 4F,G). The comparison with unspecific inhibition (global balance) for which inhibitory weights are swapped with interneurons specialized to the losing group confirms that precise timing between excitation and inhibition is important for the response sharpening (red curves versus green curves in Figures 4F,G). As in the SFC, the response to τ_in_ in the range of 1–5 ms benefits most from the detailed balance (Figure 4G).

To test the robustness of the FFC against noise, we modified the FFC by adding random uncorrelated inputs into the interneurons and the output neuron, and decreased the number of inputs from the correlated pathways (Noisy Full Feedforward Circuit, Noisy FFC; Supplementary Figure 2A). Detailed balance emerged as in the FFC, inhibitory synapses showed delay-dependent potentiation (Supplementary Figure 2B), and the response from the output neuron was sharpened (Supplementary Figures 2C,D, red curve). Detailed balance and the sharpening role of inhibition are therefore robust against noise.

We conclude that in the more realistic FFC and Noisy FFC, eSTDP determines the specialization of both the output neurons and the interneurons, and anti-Hebbian iSTDP selects the interneurons with intermediate delays, which leads to sharpening of the response.

## 3. Discussion

This study showed how eSTDP and iSTDP can jointly structure synapses in feedforward neural circuits to control downstream firing. We found that the temporally anti-Hebbian (post-pre LTP) component of iSTDP is crucial to achieve a balance between excitatory and inhibitory weights given correlated inputs, and assuming an inhibitory delay in the order of a few milliseconds. Moreover, interneurons can be recruited by Hebbian eSTDP in a self-organized fashion to develop inhibition through iSTDP onto output neurons. By selecting adequate delays in this disynaptic inhibition scheme, iSTDP sharpens the output firing response, enhancing the propagation of spike volleys.

### 3.1. Input timing and types of iSTDP learning window

We investigated how the interplay between eSTDP and iSTDP shapes the excitatory and inhibitory weight distributions. In our model, correlations in inhibition follow correlations in excitation by a delay of up to 10 ms (Figures 1, 2, and 4), which agrees with experimental observations at the order of a few milliseconds in the auditory (Wehr and Zador, 2003) and somatosensory cortices (Gabernet et al., 2005). For such input signals, we found that both anti-Hebbian and symmetric iSTDP windows generate a detailed balance between excitatory and inhibitory weights (see SFC in Figure 2). In contrast, Hebbian iSTDP leads to the weakening of all synapses: Due to the inhibitory delay and the timescale of the input correlations, a large portion of the inhibitory spikes fall into the LTD part of the window.

There is, to our knowledge, currently no experimental evidence of this kind of anti-Hebbian iSTDP. Some studies show evidence of anti-Hebbian STDP in excitatory synapses in the electric fish (Han et al., 2000; Harvey-Girard et al., 2010), in the dorsal cochlear nucleus (Tzounopoulos et al., 2004), and in corticostriatal synapses (Fino et al., 2009). Anti-Hebbian STDP has also been the subject of theoretical studies (e.g., Roberts and Bell, 2000; Rumsey and Abbott, 2004, 2006; Carnell, 2009), but again only in the context of excitatory synapses. Our study is the first one to show a functional role for the anti-Hebbian LTP in iSTDP. Anti-Hebbian LTP is also part of the symmetric iSTDP learning rule that is the subject of a recent theoretical iSTDP study by Vogels et al. (2011), showing the exact balancing of excitation and inhibition. In our model, the output neuron is dominated by strong inhibition after learning, meaning that the balance between excitatory and inhibitory weights leads to a different firing regime than in their results (Vogels et al., 2011). This follows because of our choice of inputs, which induces strong LTP via iSTDP.

Vogels et al. (2011) showed that symmetric iSTDP can lead inhibitory feedforward connections to detailed balance with fixed excitation, by letting inhibition adapt to the firing rate of each input pathway. We propose that iSTDP can ensure pathway-specific balance between excitation and inhibition, even if firing rates are constant and excitation is growing simultaneously with eSTDP. Since symmetric iSTDP contains an anti-Hebbian element (namely, post-pre LTP), detailed balance will follow as long as there is a positive delay in the inhibitory input (e.g., Pouille and Scanziani, 2001; Wehr and Zador, 2003). Our theoretical results show that the expected increase in weights does not depend on input firing rate. If, however, firing rates are unequal between input groups, we still expect our current results to hold, as long as spike pairing-based effects dominate those coming from the rate differences.

Our findings are in contrast with Hebbian iSTDP, which has been found experimentally in the entorhinal cortex (Haas et al., 2006) and in the ventral tegmental area (Kodangattil et al., 2013). If sufficient inhibition from other sources is present, synapses corresponding to uncorrelated inputs may be potentiated by Hebbian iSTDP, leading to a “reversed detailed balance”; a scenario in which inhibitory inputs from all but one pathway make up for the excitatory input from the remaining pathway. Although Hebbian iSTDP does not directly support detailed balance in the weights in our model, Hebbian iSTDP may subserve alternative functions in neural circuit processes. Recent theoretical work has shown that Hebbian iSTDP leads to decorrelation of inhibition with respect to excitation, which results in global balance and increased sensitivity to excitatory correlations (Luz and Shamir, 2012). This follows because of the increased sensitivity to input fluctuations when the neuron acts as a coincidence detector, in contrast to the integrator regime (Hong et al., 2012). Another study showed that Hebbian iSTDP also decorrelates spike patterns through lateral connections (Savin et al., 2010). Though these studies indicate that Hebbian iSTDP plays a part in creating global balance, it does not lead to the detailed balance in our feedforward circuit. Alternatively, detailed balance by Hebbian iSTDP may arise if inhibitory delays are negative, for instance when somatic inhibitory inputs precede the excitatory dendritic spike. Inhibitory weight increase will, however, be strongly bounded by the fact that an early inhibitory spike may prevent a postsynaptic spike otherwise caused by late excitation, preventing weight increase.

Another form of inhibitory plasticity, slightly different from iSTDP considered here, is voltage-dependent iLTP (Maffei et al., 2006), which leads to a potentiation in inhibitory synapses when a presynaptic spike precedes a postsynaptic depolarization either without spikes (Maffei et al., 2006), or accompanied by low-frequency spiking (Wang and Maffei, 2014). Modeling approaches have shown that when iLTP is complemented by a homeostatic form of LTD, it is capable of creating sparseness in activation that supports stimulus-pair specificity in recipient neurons (Bourjaily and Miller, 2011a,b). iLTP contains a competitive effect for inhibitory synapses, meaning that the weakest synapses will not manage to decrease post-synaptic firing, therefore missing out on LTP. If the postsynaptic spiking rate is low, as in our study, we expect the inhibitory weight evolutions with iLTP to behave similarly to Hebbian iSTDP without the LTD part. This would not lead to detailed balance, because of the brief delay in inhibition, but global balance might ensue when implemented in a large network.

In view of the large diversity of inhibitory interneurons (Markram et al., 2005), explaining the possible roles of iSTDP in different circuits and interneurons is an important open question that requires further work.

### 3.2. Recruitment of disynaptic inhibitory pathway in feedforward network

In our Full Feedforward Circuit model (FFC model), the excitation–inhibition structure in synaptic weights arises from the recruitment of interneurons: specialization due to eSTDP, followed by the strengthening of inhibition onto output neurons induced by iSTDP. Hebbian eSTDP provides a sufficient degree of temporal correlation between the selected excitatory and inhibitory pathways onto the output neuron. This correlation is essential for anti-Hebbian iSTDP to select weights from adequate interneurons, whose firing is correlated with the output neuron.

One could also imagine other combinations of eSTDP-iSTDP for the interneurons in the FFC model. For example, if the eSTDP onto the interneurons is anti-Hebbian, excitation and inhibition onto the output neuron become *anti*-correlated. We expect that Hebbian iSTDP for the inhibitory synapses from the interneurons would be an interesting choice in this case, to further reinforce the anticorrelation between excitation and inhibition onto the output neuron.

### 3.3. Control of correlated firing activity

In the feedforward circuit, iSTDP enables the neuron to select inhibition with an adequate delay (Figure 4D), which temporally controls the propagation of the volley of correlated spikes without arriving too early to stop it entirely (Figure 3D: top; Figures 3F,G). Moreover, the selected suitable delays depend on the input temporal precision (τ_in_): for temporally broader spike volleys, larger delays are recruited (Figure 4D). In this sense, the output firing is sharpened only after sufficiently many inputs have been integrated, in agreement with experimental findings (Gabernet et al., 2005).

It is worth noting that delayed inhibition compared to excitation arises naturally because of the disynaptic pathway (axonal delays of interneurons). For the inputs, we considered sharp correlations at the scale of a few milliseconds, in line with the timescale of input correlations for which neurons in a balanced state are most sensitive, shown both experimentally *in vitro* and theoretically by Rossant et al. (2011). Propagation of spike volleys in networks also requires such fine temporal resolution (Diesmann et al., 1999). Our results suggest that interneurons can control the temporal spread of such spike volleys by adapting an inhibitory cutoff. This function of iSTDP is complementary to the homeostatic stabilization (Pouille et al., 2009) enforced by iSTDP to control the average firing of neurons, as was demonstrated recently (Vogels et al., 2011). In addition to restraining the firing rate, iSTDP can control the temporal output by creating a detailed balance in the synaptic weights, in which precisely timed inhibition limits the output spikes to a narrow temporal window. Thus, our finding is in accordance with previous studies that show that inhibition limits the time for summation and integration of EPSCs (Pouille and Scanziani, 2001; Gabernet et al., 2005). The presence of inhibition improves frequency tuning in excitatory neurons in auditory cortex (Wu et al., 2008). We showed that sharpening of the response only takes place if the inhibitory delay is sufficiently brief. Such short delays can limit the range of intensity tuning in auditory neurons by reducing the EPSP amplitude, controlling the response integration window (Wu et al., 2006). Quick but delayed inhibition after excitation therefore allows only inputs from high intensities to generate spikes in the downstream neuron. The millisecond-range sharpening of the response by inhibition, such as in our model, may therefore be useful for tuning control of a neuron.

For certain delays and τ_in_, the well-timed inhibition may hyperpolarize the neuron so strongly that the responses exhibit a rebound, after inhibition vanishes (Supplementary Figure 3). This is because the strong hyperpolarization brings the membrane potential far from the excitatory reversal potential, temporarily boosting subsequent excitatory inputs. We only found rebound responses for anti-Hebbian-based iSTDP, for which inhibitory weights grew strongest. Mechanisms to regulate inhibitory strength within a medium range could prevent this phenomenon, such as an *ad hoc* upper bound on inhibitory weights or weight-dependent iSTDP.

Finally, we also showed that response sharpening was robust to noise in the circuit, even when the correlated inputs were decreased, meaning that our results can be extended to more realistic circuit contexts with larger input numbers. iSTDP and its resulting structure in weights may therefore be useful for the propagation of transient activities in larger circuits, such as a cortical column.

## 4. Materials and methods

Here we provide details about our analysis to predict the weight changes induced by simultaneously occurring eSTDP and iSTDP in the first section of Results. Then we describe the two neural circuit architectures used in this study, namely the SFC and FFC. Finally, we explain how the PSTHs of the postsynaptic and output neurons are calculated.

### 4.1. Theoretical analysis of the weight evolution in the simplified feedforward circuit (SFC)

In our theoretical model, a postsynaptic Poisson neuron post receives both excitatory and inhibitory inputs (Figure 1A). All inputs share the same source of correlation, and inhibition is delayed by *d* compared to excitation.

The firing rate ρ_post_ evolves over time according to the presynaptic inputs:

(4) $$\rho_{\text{post}}(t)=\sum_{k}w_{k}^{\text{e}}\left[\epsilon_{\text{e}}*S_{k}^{\text{e}}\right](t)-\sum_{m}w_{m}^{\text{ i}}\left[\epsilon_{\text{i}}*S_{m}^{\text{i}}\right](t).$$

The *k*th excitatory input spike train *S*^e^_*k*_ is modeled as a time series of Dirac functions: *S*^e^_*k*_ (*t*) = ∑_*s*_ δ(*t* − *t*^*k*^_*s*_); likewise, *S*^i^_*m*_ is the *m*th inhibitory spike train. Though ρ_post_ may take on negative values in theory, we assume it is positive on average, and do not consider the case of no postsynaptic spiking. The EPSPs and IPSPs are summed together to obtain ρ_post_; * denotes the convolution of functions. For each EPSP at synapse *k*, the time course of the postsynaptic response for a single spike is described by the normalized kernel functions ϵ_e_ rescaled by the weight *w*^*e*^_*k*_. For IPSP at synapse *m*, the same holds with ϵ_i_ and *w*^i^_*m*_. In Figure 1, we use a simple exponential decay that is identical for all excitatory synapses with decay time τ_e_ = 3 ms; likewise τ_i_ = 5 ms for all inhibitory synapses.

In order to evaluate the expected weight change, we calculate the pre-post spike-time correlations for excitatory and inhibitory inputs. We consider the situation when pre-post correlations are dominated by the effect of input correlations. Actually, we use spike-time covariances defined as (Gilson et al., 2010)

(5) $$\begin{array}{l} C_{k,l}^{\text{e},\text{e}}(t,\Delta t)=\text{Cov}[S_{k}^{\text{e}},S_{l}^{\text{e}}](t,\Delta t):=\langle S_{l}^{\text{e}}(t)S_{k}^{\text{e}}(t+\Delta t)\rangle \\ \;-\langle S_{l}^{\text{e}}(t)\rangle \langle S_{k}^{\text{e}}(t+\Delta t)\rangle . \end{array}$$

The angular brackets 〈 … 〉 denote the ensemble average over the randomness from the stochastic process. Considering spike trains with constant average firing rates and fixed pair-wise correlations, we can omit the dependence on *t* in Equation (5). For the configuration described in Figure 1A, excitatory inputs are homogeneously correlated between them, as well as inhibitory inputs. However, the correlation between an excitatory and an inhibitory inputs involves the delay *d*. Denoting by *C*_0_(Δ *t*) the homogeneous covariance corresponding to Figure 1C, we have

(6) $$\begin{array}{l} C_{k,l}^{\text{e},\text{e}}(\Delta t)=C_{\text{0}}(\Delta t),\\ C_{m,n}^{\text{i},\text{i}}(\Delta t)=C_{\text{0}}(\Delta t),\\ C_{k,m}^{\text{e},\text{i}}(\Delta t)=C_{\text{0}}(\Delta t-d). \end{array}$$

All covariances are defined in a similar manner to Equation (5). For the *k*th excitatory input, the covariance Cov^e^_*k*,post_ is given by the input covariance on which the postsynaptic response (EPSPs-IPSPs) operates:

(7) $$\begin{array}{l} C_{k,\text{post}}^{\text{e}}(\Delta t):=\text{Cov}[S_{k}^{\text{e}},S_{\text{post}}](t,\Delta t),S_{\text{post}}\propto \rho_{\text{post}}\\ \;=\text{Cov}\left[S_{k}^{\text{e}},\left(\sum_{l}w_{l}^{\text{e}}\epsilon_{\text{e}}*S_{l}^{\text{e}}-\sum_{n}w_{n}^{\text{i}}\epsilon_{\text{i}}*S_{n}^{\text{i}}\right)\right](\Delta t)\\ \;=\sum_{l}w_{l}^{\text{e}}[C_{k,l}^{\text{e},\text{e}}*\epsilon_{\text{e}}](\Delta t)-\sum_{n}w_{n}^{\text{i}}[C_{k,n}^{\text{e},\text{i}}*\epsilon_{\text{i}}](\Delta t)\\ \;=\sum_{l}w_{l}^{\text{e}}[C_{\text{0}}*\epsilon_{\text{e}}](\Delta t)-\sum_{n}w_{n}^{\text{i}}[C_{0}*\epsilon_{\text{i}}](\Delta t-d). \end{array}$$

The subsequent STDP weight update is given by the integral value of the learning window *W*_e_(*u*) with the pre-post covariance *C*^e^_*k*,post_(−*u*), which yields:

(8) $$\begin{array}{l} \Delta w_{k}^{\text{e}}=[C_{k,\text{post}}^{\text{e}}*W_{\text{e}}](0)\\ \;=\sum_{l}w_{l}^{\text{e}}[C_{\text{0}}*\epsilon_{\text{e}}*W_{\text{e}}](0)-\sum_{n}w_{n}^{\text{i}}[C_{\text{0}}*\epsilon_{\text{i}}*W_{\text{e}}](d) \end{array}$$

Similarly, the pre-post covariance and the expected change for the *m*th inhibitory weight is given by:

(9) $$\begin{array}{l} C_{m,\text{post}}^{\text{i}}(\Delta t)=\sum_{l}w_{l}^{\text{e}}[C_{\text{0}}*\epsilon_{\text{e}}](\Delta t+d)-\sum_{n}w_{n}^{\text{i}}[C_{\text{0}}*\epsilon_{i}](\Delta t),\\ \;\Delta w_{m}^{\text{i}}=\sum_{l}w_{l}^{\text{e}}[C_{\text{0}}*\epsilon_{\text{e}}*W_{\text{i}}](-d)\\ \;-\sum_{n}w_{n}^{\text{i}}[C_{\text{0}}*\epsilon_{\text{i}}*W_{\text{i}}](0) \end{array}$$

These formulas are used to generate Figure 1F.

### 4.2. Details of the simulated SFC

In Figures 2, 3, the SFC consists of a single postsynaptic neuron that receives a total of 200 excitatory and 50 inhibitory inputs. Half of each set of inputs consist of weakly correlated spike trains, whereas the remainder consists of random Poisson spike trains (Figure 2A).

We use a function in Brian Simulator to generate correlated spike trains, which is based on the first method in Brette (2008). The principle of this function is that a doubly stochastic process (or Cox Process) with an average (spike) rate *r*, underlies a group of inhomogeneous Poisson processes which have rates that fluctuate around *r*. Final spike trains are derived from these inhomogeneous Poisson processes, and will appear to be homogeneous, correlated spike trains with stationary rate *r*. These correlated spike trains do not have Poisson statistics, because their autocovariance is modulated by their correlation. In order to have exponential cross-correlation functions (CCF) between these spike trains, the function employs the Ornstein–Uhlenbeck process. The time-constant of the exponential CCF is a parameter called τ_*c*_ in Brette (2008) and in Brian Simulator. We focus on the standard deviation of the latencies in input spike volleys (representing input stimuli), τ_in_, where τ_in_ = τ_*c*_$\sqrt{2}$. We apply correlation strength *c* = 0.1 and CCF standard deviation τ_in_ in the range of 0.71–5.66 ms. Correlated inhibition is delayed by *d* ms. All inputs have the same firing rate *r*_in_ = 5 sp/s.

The postsynaptic neuron is a conductance-based leaky integrate-and-fire (LIF) model. Its membrane potential *V* obeys:

(10) $$\tau_{\text{m}}\frac{dV}{dt}=E_{\text{leak}}-V+g_{\text{e}}(E_{\text{e}}-V)+g_{\text{i}}(E_{\text{i}}-V)$$

With synaptic conductances *g*_*e*_ and *g*_*i*_, that decay exponentially with conductance trace parameters τ_*e*_ and τ_*i*_:

(11) $$\tau_{\text{e}}\frac{dg_{\text{e}}}{dt}=-g_{\text{e}},\tau_{\text{i}}\frac{dg_{\text{i}}}{dt}=-g_{\text{i}}$$

For every excitatory spike from synapse *k*, *g*_e_ is increased by *w*^e^_*k*_, and for every inhibitory spike from synapse *m*, *g*_i_ by *w*^i^_*m*_. Intrinsic time constants of the neuron are not considered. All simulations are run with BRIAN, a python-based neural simulator (Goodman and Brette, 2008). The simulations last 2500 s each. For plots with error bars and color maps, 10 trials are repeated for the same simulation protocol with each set of values for *d* and τ_in_. All SFC variables and parameters are listed in Table 3.

**Table 3:** **SFC and FFC variables and parameters**.

**Theoretical SFC variables**	**Description**	**Value**
ρ_post_	Postsynaptic neuron firing rate	
*s*^*e*^_*k*_, *s*^*e*^_*l*_	Presynaptic excitatory spiketrain *k* or *1*	
*S*^*i*^_*m*_, *S*^*i*^_*n*_	Presynaptic inhibitory spiketrain *m* or *n*	
ε_e_	Synaptic conductance decay function for excitation	
ε_i_	Synaptic conductance decay function for inhibition	
*w*^*e*^_*k*_, *w*^*e*^_*l*_	Synaptic weight of excitatory input *k* or *1*	
*w*^*i*^_*m*_, *w*^*i*^_*n*_	Synaptic weight of inhibitory input *m* or *n*	
*u*	Time between two spikes	
τ_e_	Excitatory conductance decay constant	3 ms
τ_i_	Inhibitory conductance decay constant	5 ms
*d*	Inhibitory delay	2–20 ms
τ_c_	Time constant for input spike correlogram	0.5–4.0 ms
τ_in_	STD of the latency of the input spike correlogram	τ_*c*_ × $\sqrt{2}$
*c*	Correlation	0.1
**NUMERICAL EXTRA VARS: SFC and FFC**		
V	Postsynaptic neuron membrane voltage	
g_*e*_	Excitatory synaptic conductance	
g_*i*_	Inhibitory synaptic conductance	
**NUMERICAL EXTRA PARAMS: SFC and FFC**		
r_*in*_	Input firing rate	5 Hz
τ_m_	Membrane time constant	20 ms
τ_i_	Inhibitory conductance decay constant	20 ms
E_leak_	Leak Potential	−70 mV
E_*e*_	Excitatory reversal potential	0 mV
E_*i*_	Inhibitory reversal potential	−80 mV
**–**	LIF spike threshold	−50 mV

Table listing all SFC and FFC variables and parameters relating to inputs and neuronal properties.

### 4.3. Details of the simulated FFC

In Figure 4, the FFC model incorporates 50 inhibitory interneurons which receive the same excitatory inputs as the output neuron, and project inhibitory connections onto the latter. In contrast to the SFC, two groups of correlated inputs compete against each other (dark blue and light blue lines in Figure 4A). The postsynaptic neuron receives 100 inputs from each group, and each interneuron receives 10 excitatory synapses from each group. The inputs are chosen so that the first interneuron receives excitatory input from spike trains 1–10 from the dark blue group, the second interneuron receives input from spike trains 2–11 from the dark blue group, and so on. The same procedure is performed for inputs from the light blue group. The 50 interneurons only differ from the output neuron by a shorter membrane time constant τ^i^_m_ = 5 ms. The interneurons are not connected to one another and there is no external inhibition source. Each interneuron makes a single inhibitory synapse onto the output neuron, and axonal delays *d* are heterogeneous, ranging from 0 to 9 ms (five interneurons for each *d*).

All synapses are plastic. The excitatory synapses onto both the output neuron and the inhibitory interneurons are subject to the same Hebbian eSTDP leaning window. The list of parameters that vary from the SFC is shown in Table 2. The iSTDP window time constants are lower (τ^i^_pre_ = τ^i^_post_ = 20 ms), excitatory and inhibitory learning are slowed down (η_e_ = 0.0624, η_i_ = 0.02), the eSTDP equilibrium value is higher (*w*_0_ = 0.08), and the value of the start-up weights is changed (a random number between 0 and 1 for excitation, 1 for inhibition). Total simulation time is 1000 s.

To test the robustness against noise, we modify the FFC by adding 400 excitatory random inputs onto the interneurons and output neuron (Noisy FFC in Supplementary Figure 2A, green inputs) and decrease the size of the correlated groups to 50 (dark and light blue inputs). The number of interneurons is increased to 120. Other parameters are unchanged (see Table 3 for all FFC parameters).

### 4.4. Analysis of the temporal acuity of the postsynaptic response

We evaluate how the iSTDP learning rule, via the resulting weight distributions, shapes the postsynaptic response to correlated input activity. To do so we run the SFC simulation with fixed weights for 300 s.

Volleys of input spikes (“events”) are detected by binning the spike times of the 100 correlated inputs in the SFC in bins with width 0.5 ms, and counting the spikes in a sliding window of duration τ_in_. When the spike count exceeds a threshold, the time of the event is set to the center of the sliding window. Events in neighboring windows are discarded. The window spike count threshold is determined for each τ_in_ such that the average number of events per second is as close as possible to *r*_in_ without exceeding it.

To evaluate the temporal acuity of the spikes fired in response to such events, we count the postsynaptic spikes in bins of 0.5 ms. This yields a peri-stimulus time histogram (PSTHs) around the time of the events, as shown in Figure 3B. We then evaluate the temporal acuity of the response of the output neuron to input stimuli by computing the sharpness of the PSTH.

Not only the latencies of spikes following the event, but also the excess of spikes compared to the baseline output firing rate contributes to the temporal acuity of the response. We obtain the average firing rate during the entire 300 s simulation, *F*_0_. The number of spikes in each bin of the PSTH is then divided by *F*_0_, yielding the “normalized” PSTH as a deviation from average activity. This deviation is also the signal to noise ratio (SNR: Figures 3C1,C2). The temporal acuity of the response input events is then evaluated through the standard deviation of the normalized PSTH, τ_out_. τ_out_ is computed over the time window 0,+10 ms inside the PSTH (0 is the time of the event).

To study how the emerged inhibitory weight structure affects τ_out_, we compare the outcome of simulations to two controls:

- “specific inhibition”: the weights are as in the numerical simulation of the SFC;
- “unspecific inhibition control”: the inhibitory weights are swapped between the two input groups, leading to equivalent total inhibition, but abolishing the relation between the temporal structure of the spike trains and the strength of the weights that depended on them through iSTDP;
- “excitation only control”: the inhibitory inputs are removed completely, leaving only the excitatory inputs.

In the unspecific inhibition control for the SFC, we aim to destroy the detailed weight structure that emerges, but preserve strong feedforward inhibition. After swapping the inhibitory weights between the correlated and random inputs, the weight strengths are adjusted down to obtain a postsynaptic firing rate similar to the specific inhibition configuration. Weight corrections are not performed for trials with mean weight smaller than 1. Excitatory weights are unchanged in all three conditions. In the FFC, the same three scenarios are applied. For specific inhibition, all weights are as obtained from the simulation. To obtain the unspecific inhibition condition, excitatory weights onto the interneurons are swapped between the winner and loser input pathways. The result of this manipulation is that an interneuron receiving strong inputs from the dark blue group and weak inputs from the light blue group, changes to receiving weak inputs from the dark blue group and strong inputs from the light blue group. This procedure leads to qualitatively the same control as in the SFC. Inhibitory weights are not adjusted further as in the SFC. For the excitation only control, the interneurons are omitted.

## Author contributions

In this study, Matthieu Gilson and Florence I. Kleberg designed the experiments, Florence I. Kleberg performed the experiments and analyzed the data, and Florence I. Kleberg, Matthieu Gilson, and Tomoki Fukai wrote the paper.

## Funding

The research was funded by RIKEN.

### Conflict of interest statement

The authors declare that the research was conducted in the absence of any commercial or financial relationships that could be construed as a potential conflict of interest.

## Supplementary material

Supplementary Figure 1 **Equilibrium weights of the random inhibitory inputs in the SFC**. Mean inhibitory weight for Hebbian iSTDP (red curve), anti-Hebbian iSTDP (magenta curve), symmetric iSTDP (black curve), and symmetric with equal total LTP (gray curve). Mean final weights are shown for three τ_in_.

Supplementary Figure 2 **Robustness of weight structure development and response sharpening in the presence of noise**. **(A)** Noisy full feedforward circuit model with eSTDP and iSTDP. There are 120 interneurons instead of 50. The correlated input groups have decreased to 50 inputs each, and an additional 400 random inputs project onto the output neuron. Each interneuron also receives 60 random inputs. For eSTDP, *w*_0_ = 0.037. Other parameters are as in the FFC. **(B)** Delay-dependent inhibitory weight strenghtening of interneurons recruited by the winning group (top) and absence of inhibitory weight increase for interneurons recruited by the losing group (bottom). **(C)** Effect of inhibition on the response of the postsynaptic neuron in response to correlated events for τ_in_ = 2.12 ms (left) and 3.54 ms (right). Comparison of the signal/noise ratio(SNR) between specific inhibition (red), the control of unspecific inhibition (green) and excitation only (black). **(D)** τ_in_ and τ_out_ results for the Noisy FFC with specific inhibition (red), unspecific inhibition (green), and with only excitation (black) for various τ_in_.

Supplementary Figure 3 **Rebound of the neuronal spike probability after the arrival of strong inhibition in the SFC**. The rebound in spiking probability is visible for specific inhibition (red curves). In the specific inhibition case, a rebound response is observed (orange arrow). The red arrow indicates the moment inhibition kicks in, in the specific inhibition case. The rebound response is shown for τ_in_ = 0.71 ms, *d* = 5, and 6 ms.
