# Supplementary material for: Excitatory and inhibitory STDP jointly tune feedforward neural circuits to selectively propagate correlated spiking activity
Source: Front Comput Neurosci. 2014 May 7;8:53. doi: 10.3389/fncom.2014.00053 (PMC4019846; doi:10.3389/fncom.2014.00053)
Supplement: Supplementary Figure 1 — Equilibrium weights of the random inhibitory inputs in the SFC. Mean inhibitory weight for Hebbian iSTDP (red curve), anti-Hebbian iSTDP (magenta curve), symmetric iSTDP (black curve), and symmetric with equal total LTP (gray curve). Mean final weights are shown for three τin. [file Presentation1.PDF]

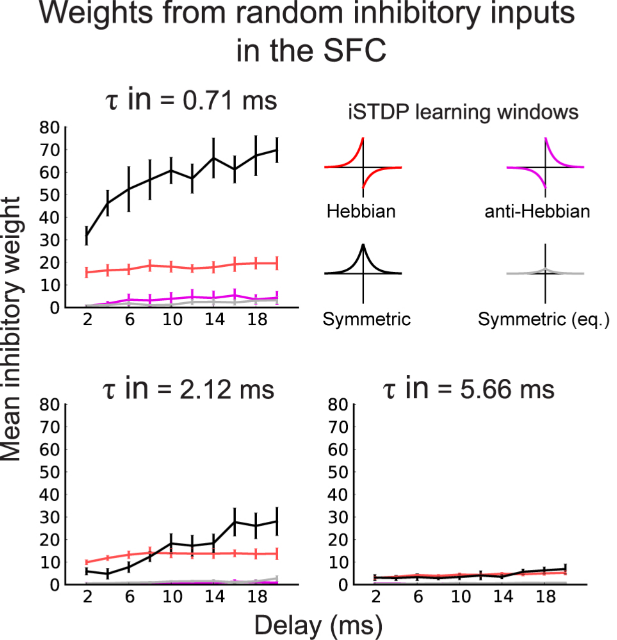

Weights from random inhibitory inputs in the SFC
τ in = 0.71 ms
iSTDP learning windows
Hebbian
anti-Hebbian
Symmetric
Symmetric (eq.)
τ in = 2.12 ms
τ in = 5.66 ms
Mean inhibitory weight
Delay (ms)
0
10
20
30
40
50
60
70
80
2
6
10
14
18
