# Supplementary figures and images for: Excitatory and inhibitory STDP jointly tune feedforward neural circuits to selectively propagate correlated spiking activity
Source: Front Comput Neurosci. 2014 May 7;8:53. doi: 10.3389/fncom.2014.00053 (PMC4019846; doi:10.3389/fncom.2014.00053)

# PSTH with rebounding response after inhibition

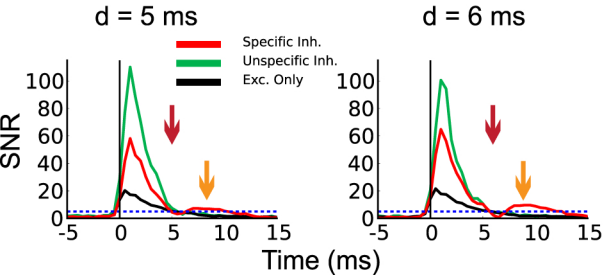

Supplement: Supplementary Figure 2 — Robustness of weight structure development and response sharpening in the presence of noise. (A) Noisy full feedforward circuit model with eSTDP and iSTDP. There are 120 interneurons instead of 50. The correlated input groups have decreased to 50 inputs each, and an additional 400 random inputs project onto the output neuron. Each interneuron also receives 60 random inputs. For eSTDP, w0 = 0.037. Other parameters are as in the FFC. (B) Delay-dependent inhibitory weight strenghtening of interneurons recruited by the winning group (top) and absence of inhibitory weight increase for interneurons recruited by the losing group (bottom). (C) Effect of inhibition on the response of the postsynaptic neuron in response to correlated events for τin = 2.12 ms (left) and 3.54 ms (right). Comparison of the signal/noise ratio(SNR) between specific inhibition (red), the control of unspecific inhibition (green) and excitation only (black). (D) τin and τout results for the Noisy FFC with specific inhibition (red), unspecific inhibition (green), and with only excitation (black) for various τin. [file Presentation2.PDF]

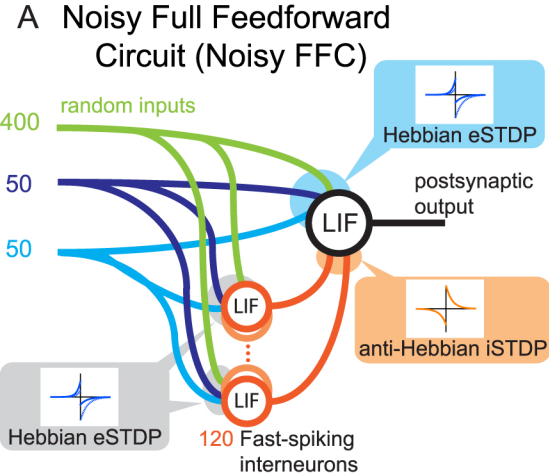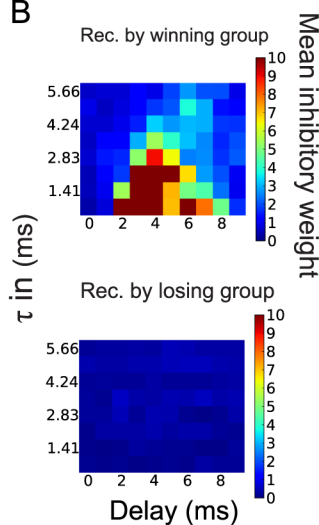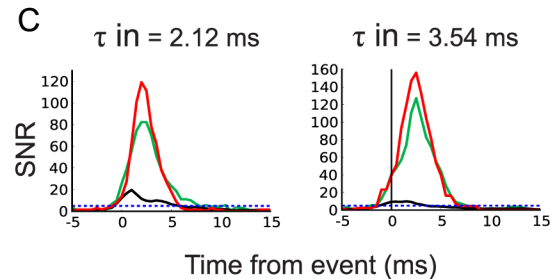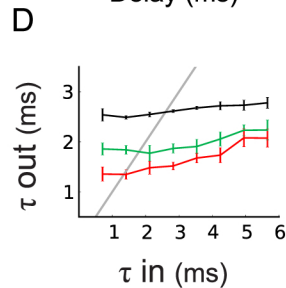

Supplement: Supplementary Figure 3 — Rebound of the neuronal spike probability after the arrival of strong inhibition in the SFC. The rebound in spiking probability is visible for specific inhibition (red curves). In the specific inhibition case, a rebound response is observed (orange arrow). The red arrow indicates the moment inhibition kicks in, in the specific inhibition case. The rebound response is shown for τin = 0.71 ms, d = 5, and 6 ms. [file Presentation3.PDF]
